# Whole Blood Interferon-Gamma Responses to *Mycobacterium tuberculosis* Antigens in Young Household Contacts of Persons with Tuberculosis in Uganda

**DOI:** 10.1371/journal.pone.0003407

**Published:** 2008-10-15

**Authors:** Deborah A. Lewinsohn, Sarah Zalwango, Catherine M. Stein, Harriet Mayanja-Kizza, Alphonse Okwera, W. Henry Boom, Roy D. Mugerwa, Christopher C. Whalen

**Affiliations:** 1 Department of Pediatrics, Oregon Health & Science University, Portland, Oregon, United States of America; 2 Uganda-CWRU, Research Collaboration, Kampala, Uganda; 3 Department of Epidemiology and Biostatistics, Case Western Reserve University School of Medicine, Cleveland, Ohio, United States of America; 4 Tuberculosis Research Unit, Case Western Reserve University School of Medicine, Cleveland, Ohio, United States of America; 5 Department of Medicine, Makerere University Medical School, Kampala, Uganda; 6 National TB and Leprosy Program, and Mulago Hospital, Kampala, Uganda; 7 Department of Medicine, Case Western Reserve University School of Medicine and University Hospitals Case Medical Center, Cleveland, Ohio, United States of America; University of Melbourne, Australia

## Abstract

**Background:**

Due to immunologic immaturity, IFN-γ-producing T cell responses may be decreased in young children compared to adults, thus we hypothesized that IFN-γ responses to mycobacterial antigens in household contacts exposed to *Mycobacterium tuberculosis* (Mtb) would be impaired in young children relative to adults. The objective of this study was to compare whole blood IFN-γ production in response to mycobacterial antigens between children and adults in Uganda.

**Methodology/Principal Findings:**

We studied household contacts of persons with culture-positive pulmonary tuberculosis (TB) enrolled in a cohort study conducted in Kampala, Uganda. Whole blood IFN-γ production in response to Mtb culture-filtrate antigens was measured by ELISA and compared between infants (<2 years old, *n* = 80), young children (2 <5 years old, *n* = 216), older children (5 <15 years old, *n* = 443) and adults (≥15 years old, *n* = 528). We evaluated the relationship between IFN-γ responses and the tuberculin skin test (TST), and between IFN-γ responses and epidemiologic factors that reflect exposure to Mtb, and the effect of prior BCG vaccination on IFN-γ responses. Young household contacts demonstrated robust IFN-γ responses comparable to those of adults that were associated with TST and known risk factors for infection. There was no effect of prior BCG immunization on the IFN-γ response.

**Conclusions/Significance:**

Young children in a TB endemic setting can mount robust IFN-γ responses generally comparable to those of adults, and as in adults, these responses correlated with the TST and known epidemiologic risk factors for Mtb infection.

## Introduction

Pediatric tuberculosis represents a major cause of childhood morbidity and mortality worldwide, affecting more than 800,000 children each year, and comprising approximately 10% of all cases of TB [1]. Approximately 75% of childhood TB cases occur in high burden countries with limited resources for health care. Infants and young children are more likely than adults to develop TB following infection with Mtb and disease is more likely to be severe as reflected in the increase incidence of miliary TB and TB meningitis [2]. The propensity for infants to develop severe TB following infection is likely, at least in part, to reflect immunologic immaturity.

Host defense to TB requires strong CD4^+^ Th1 cell responses [3]. Specifically, CD4^+^ T cell responses are critical for containment of infection in the mouse TB model and individuals with HIV infection in whom CD4^+^ T cells are depleted are susceptible to TB. CD4^+^ Th1 cells are defined by production of IFN-γ. In the murine TB model, IFN-γ is necessary for control of Mtb [3] and rare humans with mutations in the IFN-γ receptor are prone to mycobacterial infection [4]. Young infants demonstrate diminished CD4^+^ Th1 cell responses in response to pathogens compared to adults [5]. Therefore, we hypothesized that IFN-γ production in response to mycobacterial infection would be deficient compared to those observed in older children and adults. To address this hypothesis, we analyzed whole blood IFN-γ responses to Mtb antigens among household contacts of different ages in a setting of high level Mtb transmission. This analysis represents the largest study of IFN-γ responses to Mtb antigens in young household contacts of adult TB patients and the only direct comparison of young children to older children and adults.

## Results

**Figure 1:**
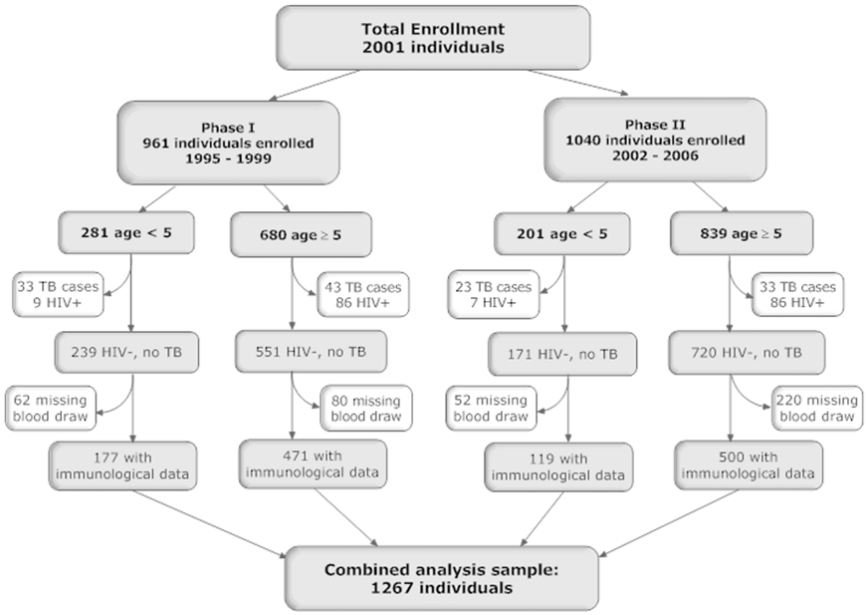
Flow diagram depicting enrollment into two consecutive household contact studies. The numbers of individuals enrolled by age, followed by elimination of individuals with TB, HIV infection, or inadequate blood specimens, resulting in the number of individuals evaluated by IFN-γ assays, are shown.

A total of 2301 household contacts were enrolled in two prospective cohorts accrued between October 1995 and April 2006. Three hundred individuals were eliminated from analysis because of incomplete clinical and laboratory testing at baseline. Of the remaining 2001 individuals, 961 individuals were enrolled from 1995–1999 (Phase I, [6]) and 1040 individuals were enrolled from 2002–2006 (Phase II, Figure 1). The two study populations were similar with regard to demographic factors, and prevalence of TB at enrollment, latent TB infection (LTBI), and HIV-infected individuals. Of the total household contacts, 482 (281 in Phase I and 201 in Phase II) were <5 years old and 1519 (680 in Phase I and 839 in Phase II) were ≥5 years old. Of total household contacts, we excluded 132 contacts with TB and 188 contacts who were HIV infected to obtain a total of 1681 healthy HIV uninfected household contacts (410 contacts <5 years old and 1271 contacts ≥5 years). Finally of these, 296 children <5 years old and 971 older children and adults had immunological data that could be evaluated. The main reason that immunologic data was unavailable was that after clinical testing there was insufficient blood for immunological assays. More children <5 years than ≥5 years had missing immunologic data (27.7% vs. 23.6%, respectively, p = 0.089) reflecting the difficulties of obtaining blood from young children. Since this study compared immune responses of infants and young children to those of older children and adults, ≥5 mm (the cut-off for a positive TST in household contacts <5 years) was used to define a positive TST throughout the analysis. A positive TST at baseline, was more common among contacts ≥15 years or older, than contacts <15 years (85% vs. 67%, p<0.0001; Table 1). A BCG scar was identified more often in children younger than 15 years than in older contacts (79% vs. 58%, p<0.0001).

**Table 1:** Baseline demographic and clinical characteristics of healthy, HIV seronegative household contacts according to age range.

	<2 years	2 <5 years	5 <15 years	≥15 years	P value^1^
	*n* = 80	*n* = 216	*n* = 443	*n* = 528	
Female sex^2^	42 (52.5%)	101 (46.8%)	239 (54.0%)	329 (62.3%)	**0.001**
TST ≥5 mm^2^	53 (66.2%)	140 (64.8%)	301 (68.0%)	449 (85.0%)	**<0.0001**
BCG scar^2^	63 (78.8%)	172 (79.6%)	347 (78.3%)	295 (55.9%)	**<0.0001**

1 Chi-square test for heterogeneity across groups.

2 Numbers of subjects, followed by the percentages of subjects relative to the total number of subjects in each age group in parentheses, are shown.

**Figure 2:**
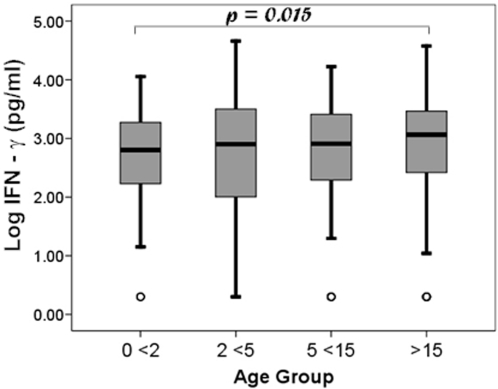
Comparison of whole blood IFN-γ responses to Mtb antigens for different age groups. Boxplots illustrate the median and distribution of log_10_-transformed IFN-γ results by age group (<2, 2 <5, 5 <15, and ≥15 years).

The magnitude and distribution of whole blood IFN-γ responses of all HIV negative household contacts at the time of study enrollment were similar in all age groups (Figure 2). The median log_10_ IFN-γ responses ranged from 2.80 among infants <2 years to 3.06 among adults. The responses were higher in household contacts ≥15 years old than infants (<2 years; p = 0.015), but the differences in medians among groups were small (less than 0.3 log units) and unlikely to be biologically significant. Even the youngest household contacts mounted IFN-γ responses comparable to older children and adults. There were no other statistically significant differences seen among age groups.

To further analyze the magnitude of the IFN-γ response across age groups, we studied the proportion of positive IFN-γ assays in comparison to the proportion of positive TSTs between different age groups. Specifically, we analyzed the concordance between IFN-γ assay results and TST results in young children (<5 years) compared to older children and adults (≥5 years, Table 2). Concordance between the IFN-γ response and the TST was statistically significant for both age groups (<5 years, κ = 0.397; ≥5 years, κ = 0.394; p<0.0001). Concordance rates between whole blood IFN-γ assay and TST results were similar in young children and older children and adults (p = 0.554).

**Table 2:** Concordance between IFN- γ assay and TST result by age of contacts.

	Age group	
	<5 years	≥5 years
**IFNγ+/TST+**	146 (49.3%)	612 (63%)
**IFNγ+/TST−**	36 (12.2%)	86 (8.9%)
**IFNγ−/TST+**	47 (15.9%)	138 (14.2%)
**IFNγ−/TST−**	67 (22.6%)	135 (13.9%)
**IFNγ+ rate**	0.615	0.719
**TST+ rate**	0.652	0.772
**Concordance rate**	0.720	0.769
**κ coefficient** 1, **(SE)**	0.397 (0.055)	0.394 (0.033)

TST = Tuberculin skin test; IFNγ = results of the whole blood IFN-γ assay for Mtb antigen; SE = Standard error. ^1^p<0.0001 for concordance between TST and IFNγ.

**Figure 3:**
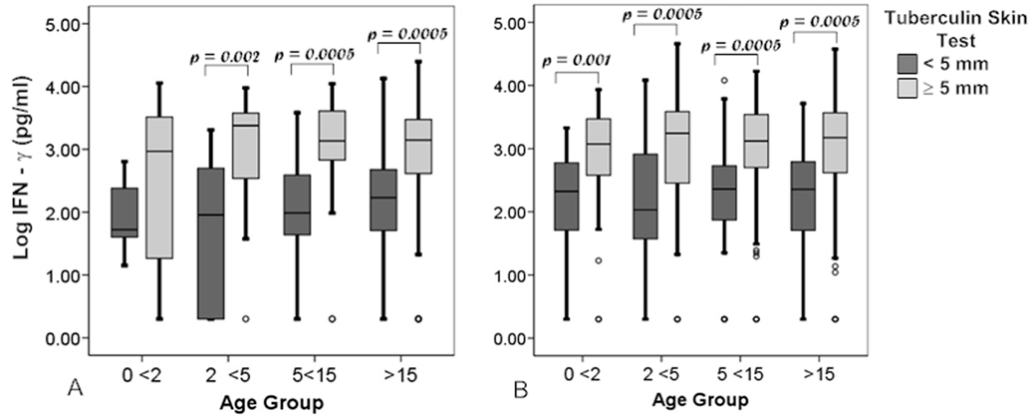
(a) Comparison of whole blood IFN-γ assay results by age and TST status in BCG unimmunized individuals. Boxplots that illustrate the median and distribution of log_10_-transformed IFN-γ results of BCG unimmunized household contacts are shown. Comparisons between age groups are shown. Additionally, within each age group, comparisons between TST positive and TST negative are shown. Numbers of individuals in each subgroups is as follows: <2, TST negative, 8; <2, TST positive, 9; 2 <5, TST negative, 18; 2 <5, TST positive, 26; 5 <15, TST negative, 29; 5 <15, TST positive, 67; ≥15, TST negative, 37; ≥15, TST positive, 146. (b) Comparison of whole blood IFN-γ assay results by age and TST status in BCG immunized individuals. Boxplots that illustrate the median and distribution of log_10_-transformed IFN-γ results of BCG immunized household contacts are shown. Comparisons between age groups are shown. Additionally, within each age group, comparisons between TST positive and TST negative are shown. Numbers of individuals in each subgroups is as follows: <2, TST negative, 19; <2, TST positive, 44; 2 <5, TST negative, 58; 2 <5, TST positive, 114; 5 <15, TST negative, 113; 5 <15, TST positive, 234; ≥15, TST negative, 42; ≥15, TST positive, 253.

To compare the relationship of the TST to IFN-γ responses, we compared the magnitude of IFN-γ responses between TST positive and TST negative individuals within each age group of healthy household contacts (Figure 3). Furthermore, to evaluate the effect of prior BCG vaccination on IFN-γ responses, we analyzed household contacts without a history of prior BCG vaccination (Figure 3A) separately from household contacts with a history of prior BCG vaccination (Figure 3B). Among BCG unimmunized individuals (Figure 3A), there were no significant differences in median IFN-γ assay results among age groups of TST negative individuals. However, within each age group, median IFN-γ responses of TST positive individuals were consistently higher than those of TST negative individuals and for all age groups except for the <2 year olds, the differences were statistically significant (<2 years, p = 0.281; 2 <5 years, p = 0.002; 5 <15 years, p<0.0005; and ≥15 years, p<0.0005). Among BCG vaccinated individuals, median IFN-γ responses of TST positive individuals were higher than those of TST negative individuals and for all age groups the differences were statistically significant (<2 years, p<0.001; 2 <5 years, <0.0005; 5 <15 years, p<0.0005; and ≥15 years, p<0.0005). Furthermore, using a linear regression model, which includes age, BCG, and TST result, the only significant predictor of log_10_ IFN-γ was TST status (TST, p<0.0005; BCG, p = 0.144; age, p = 0.161). These data suggest that the magnitude of the IFN-γ whole blood response correlates with TST in young children as it does in adults. Moreover, BCG vaccination does not appear to affect median IFN-γ responses.

Finally, we examined the relationship between IFN-γ responses and established risk factors that reflect exposure to Mtb. Among children <5 years old, cavitary disease, sputum acid fast bacillus (AFB) grade, and extent of disease on chest x-ray in the index case were associated with higher IFN-γ responses (data not shown, p<0.05). Similarly, among contacts ≥5 years old, IFN-γ responses were associated with cavitary disease and higher smear grade in the index case (p<0.05). As for household characteristics, crowding and sharing a bed were associated with higher IFN-γ responses among contacts ≥5 years old, but not in younger children (p<0.05). In general, IFN-γ levels were consistently higher for risk factors reflecting greater exposure to the index case.

## Discussion

In general, in response to infectious pathogens and vaccines, the T cell response of young children may demonstrate decreased magnitude and slower kinetics than that observed in older children and adults, and it is more likely to represent TH2-type T cell immunity [5]. More specifically regarding Mtb antigens, Kampmann and colleagues showed that IFN-γ production in response to Mtb antigens was lower in young children (<5 years old) than in older children (5–15 years old) [7]. However in this cohort of household contacts of culture-positive pulmonary tuberculosis patients from a TB endemic area, we observed that young children mounted robust IFN-γ responses generally comparable to adults. Our study provides the first direct comparison of adults and children from the same household contact setting. Moreover, adequate numbers of individuals of various ages were enrolled to allow statistically robust comparisons. Therefore, we conclude that in these healthy young children from a TB endemic area, who do not develop active TB following household exposure to Mtb, young age itself did not hamper the development of adult-like IFN-γ responses to Mtb antigens.

Prompt, accurate identification of infants and young children with Mtb infection is an important priority as they are at higher risk of progression to disease and of developing more severe disease than older children and adults [2]. Until recently, diagnosis of LTBI has relied on the tuberculin skin test (TST). However, development of interferon-gamma (IFN-γ) release assays (IGRAs) offer new ways to make the diagnosis of LTBI. One type of IGRA is the whole blood assay, which detects Mtb-antigen induced IFN-γ expression by T cells in whole blood using an ELISA (QuantiFERON®-TB Gold and QuantiFERON®- Gold IT [Cellestis, Corp]). A second type of IGRA is based on the ELISPOT assay which detects the frequency of IFN-γ secreting cells in response to Mtb antigens (T-spot.*TB* test [Oxford Immunotec, Inc.]). Although these assays are technical and expensive, there is interest in developing them further for use in resource-limited settings where TB is endemic. The usefulness of IGRAs in making the diagnosis of Mtb infection in children is not yet firmly established and whether or not age itself, due to a decreased capacity to produce IFN-γ, decreases the sensitivity of IGRAs remains controversial. For example, the QuantiFERON®-TB demonstrated poor sensitivity for diagnosis of LTBI in Australian children, and negative results correlated with young age [8]. Furthermore, a study of IGRAs in a diverse clinical population showed that indeterminate results of the QuantiFERON Gold test were disproportionately high in children less than 5 years of age [9]. However, one study of South African children, found that ELISPOT was more sensitive than TST in diagnosing Mtb infection with children <3 years of with probable or confirmed TB [10] and a study of European children showed that both QuantiFERON Gold and T-spot.*TB* were as sensitive as TST in detecting Mtb infection in children with culture confirmed TB [11]. One implication of our results for the potential utility of IGRAs in children is that the results of this large study of household contacts from a TB endemic, where the immune responses of children of all ages and adults were directly compared, indicates that IFN-γ responses in healthy individuals were equivalent and robust, and hence the sensitivity of IGRAs should not be compromised by young age alone.

IFN-γ responses in young children, as in adults, correlated with the TST and known risk factors for infection. Our results are consistent with those from child household contact studies using an ELISPOT assay [12], [13] or a whole blood assay [14], [15] where IFN-γ responses were also correlated with TST results and risk factors for infection. The concordance rates between commercial IGRAs and TST have been studied in several studies of children and vary widely from concordance rates of 26% to 95% (κ coefficients = 0.08–0.73) depending upon the clinical setting [8], [11], [14]–[18]. We found a concordance rate (72%) which falls within this broad range. Moreover, in our study, we directly compare concordance between young children and adults in the household contact setting and found them to be similar.

Our analysis of children enrolled in the first two years of the first household contact study [19] as well as the current analysis of children enrolled over both studies presented here suggest that, in a setting of high exposure to Mtb within an endemic community, prior BCG vaccination does not affect TST results. Moreover, we conclude that prior BCG vaccination does not affect IFN-γ results. This is different than the results of Soysal et al [12] who reported that their in-house IFN-γ ELISPOT assay was negative more often in BCG immunized than in unimmunized children. It is possible that differences in methodology could account for the discrepancy in the results between these two studies in that in the Soysal et al study, an ELISPOT assay using short-term overnight incubation and Mtb specific antigens, ESAT-6 and CFP-10 were used while in our study a longer term (5–7 day) whole blood assay with Mtb culture filtrate antigens were used.

The conclusions of our study of healthy young Ugandan household contacts should not be extrapolated to young children immunocompromised by conditions other than age such as HIV. Future studies, including those using commercially available IGRAs, should continue to elucidate the interaction between the young host, Mtb and distinct immunocompromising conditions. In addition, while young children are capable of mounting strong IFN-γ responses to Mtb antigens, it remains to be determined whether or not difference in other aspects of the T cell response to Mtb could predispose children to development of active TB, such as excessive Th2, Th17, and/or T regulatory responses and/or deficient CD8^+^ T cell responses. Alternatively, perhaps differences in innate, rather than adaptive immunity could be responsible for these differences in disease susceptibility, all areas which will require addition study.

## Materials and Methods

### Patients and inclusion criteria

This study was performed in healthy, HIV seronegative household contacts of adult pulmonary TB cases enrolled in one of two prospective cohorts accrued between October 1995 and April 2006 in Kampala, Uganda [6], [20]. The enrollment criteria for the two cohorts were similar except that the earlier cohort enrolled only smear positive adult pulmonary TB cases whereas the second cohort enrolled culture positive TB index cases that could be sputum smear negative or positive.

Subjects were enrolled after institutional review board approval was secured at both University Hospitals Case Medical Center in Cleveland and the Uganda Council for Science and Technology in Uganda, and written informed consent was obtained from the head of the household and all individuals within the household. At baseline, evaluation for active TB was performed in all household contacts with symptoms consistent with TB and in all children less than 5 years of age whether symptomatic or not. All subjects were followed prospectively for two years and evaluation for active TB was repeated in all individuals who developed symptoms consistent with TB during this time. Evaluation included a medical examination, sputum microscopy, mycobacterial culture of sputum and for children, gastric aspirates, and chest radiograph. HIV testing was performed after consent and counseling using enzyme-linked immunosorbent assay. For children, HIV testing was done on symptomatic children <10 years old and healthy children <2 years old born to HIV seropositive women, otherwise the child was considered to be HIV seronegative. For children ≤18 months old with a HIV seropositive mother, HIV was excluded by HIV DNA PCR when available. Vaccination with BCG was determined by the presence of a scar on the left deltoid and verified using medical records when possible. TST was performed at baseline on all household contacts and starting in 2002, was performed serially on TST negative individuals using purified protein derivative (PPD, 5 TU, Tubersol; Connaught Laboratories, Limited, Toronto, Canada) and the Mantoux method. A positive TST was defined as ≥5 mm in children <5 years of age and in HIV infected individuals and was defined ≥10 mm in HIV uninfected individuals ≥5 years of age. All subjects <5 years old and all HIV infected subjects in whom active TB was ruled out received preventive therapy with isoniazid (INH) regardless of TST result. Starting in 2002, HIV uninfected individuals ≥5 years of age with a positive TST at baseline or who converted their TST from negative to positive during the two year follow-up period were offered preventive therapy with INH. LTBI treatment decisions were based solely on age, HIV serology and TST criteria.

From all enrolled household contacts with baseline clinical, HIV, and TST evaluation, individuals with TB at baseline or who developed TB at any point during the two year follow-up, individuals in whom HIV testing was positive or not performed, and individuals without immunologic data at baseline were removed from the analysis, as shown in Figure 1. The resulting study group comprised household contacts who were HIV seronegative, had active TB ruled out through clinical and laboratory evaluation, and had a whole blood IFN-γ assay performed at the time of enrollment. Groups for analysis were defined by age (<2 years; 2 <5 years; 5 <15 years; and ≥5 years or older) which were based on observed risk of disease (<5 years) and increased severity of disease (<2 years) in household contacts of infectious tuberculosis cases [1], [6]. For this analysis, adults were defined as individuals 15 years of age or older. TST results reported herein were those obtained from the TST placed at enrollment.

### IFN-γ release assay

For all data reported herein, a whole blood IFN-γ assay was performed at baseline. Whole blood was collected via venipuncture prior to the placement of the TST and placed in a heparinized tube. Within four hours, whole blood was diluted 1∶10 with RPMI 1640 and cultured in 48-well tissue culture plates (1 ml/well) with or without Mtb culture filtrate (10 µg/ml) and incubated at 37° for 5–7 days at which point supernatants from cultured cells were collected and cryopreserved (−70°C) for batch testing by ELISA. IFN-γ concentration (pg/ml) in culture supernatants was determined with a commercial ELISA (Endogen, Rockford, IL) in duplicate wells according to the manufacturer's instructions. We defined a positive IFN-γ assay as exceeding 304 pg/ml. This cut-off value was derived as the sum of the mean plus two standard deviations derived from all the media wells. For assays performed on subjects enrolled in the first cohort, no PHA control was included. For assays performed on subjects enrolled in the second cohort, a PHA control (5 µg/ml) was included. Mtb culture filtrate was obtained from the TB Vaccine Testing and Research Materials Contract (HHSN266200400091C, NIH, NIAID, P.I., John Belisle) or from Robert Wallis (UMDNJ-New Jersey Medical School, Newark, NJ) and prepared as described (http://www.cvmbs.colostate.edu/microbiology/tb/mixculturefilp.htm). Briefly, Mtb was grown to late-log phase in glycerol-alanine-salts medium. The culture supernatant was filtered, concentrated by ultrafiltration, and dialyzed against 0.01 M ammonium bicarbonate.

### Statistics

Comparisons of demographic and clinical characteristics according to age range were performed using a chi-square test for heterogeneity across groups. For results of whole blood IFN-γ assays, IFN-γ levels were transformed using the logarithm base-10 (log_10_) to reduce variance in distributions. As PHA positive control data was limited to whole blood assays performed on participants in the second cohort, we performed a preliminary analysis to estimate the percentage of assays in which viable functional cells were tested, i.e. interpretable assays. Specifically, an interpretable assay was defined as one in which either the response to Mtb antigen was positive (as defined below) or the PHA response was positive (>50 pg/ml). Using these criteria, 544 of 570 (95%) of all assays in which the PHA control was performed were interpretable. In addition, the proportion of un-interpretable assays was not statistically significantly different between individuals <5 years and those ≥5 years (7.2% versus 3.9%, p = 0.159). Therefore, we chose to include all available whole blood assay results on all eligible individuals regardless of whether or not the PHA control was included in the assay. For comparisons of IFN-γ as a continuous variable, non-parametric methods (Mann-Whitney test) were used for comparisons because the distribution of cytokine levels was not normal, even after logarithm transformation. To assess the combined effects of age, TST status, and BCG vaccination on log-transformed IFN-γ, we conducted a regression analysis, utilizing a George-Elston transformation to allow for the non-normality of the data [21]. To assess the concordance between the TST and our IFN-γ assay, we determined the percentage of individuals in each category (positive TST/positive IFN-γ, positive TST/negative IFN-γ, negative TST/positive IFN-γ, negative TST/negative IFN-γ), stratified by age. The concordance rate was determined from individuals positive for both plus those negative for both divided by the total, and the discordance rate was the complement of the concordance rate. A kappa (κ) statistic was estimated to illustrate the agreement between the two tests, and a significant p-value indicates that the agreement between the two tests was not due simply to chance.
